# CD206+ M2-Like Macrophages Are Essential for Successful Implantation

**DOI:** 10.3389/fimmu.2020.557184

**Published:** 2020-10-23

**Authors:** Yosuke Ono, Osamu Yoshino, Takehiro Hiraoka, Erina Sato, Yamato Fukui, Akemi Ushijima, Allah Nawaz, Yasushi Hirota, Shinichiro Wada, Kazuyuki Tobe, Akitoshi Nakashima, Yutaka Osuga, Shigeru Saito

**Affiliations:** ^1^Department of Obstetrics and Gynecology, Teine Keijinkai Hospital, Sapporo, Japan; ^2^Department of Obstetrics and Gynecology, Kitasato University School Medicine, Tokyo, Japan; ^3^Department of Obstetrics and Gynecology, Faculty of Medicine, University of Tokyo, Tokyo, Japan; ^4^Department of Obstetrics and Gynecology, Faculty of Medicine, University of Toyama, Toyama, Japan; ^5^Department of Molecular and Medical Pharmacology, Faculty of Medicine, University of Toyama, Toyama, Japan; ^6^First Department of Internal Medicine, University of Toyama, Toyama, Japan

**Keywords:** CD206, diphtheria-toxin receptor mouse, fibroblast growth factor, implantation, M2 macrophage, Wnt/β-catenin signal

## Abstract

Macrophages (MΦs) play important roles in implantation. Depletion of CD11b+ pan-MΦs in CD11b-diphtheria-toxin-receptor (DTR) mice is reported to cause implantation failure due to decreased progesterone production in the corpus luteum. However, of the M1 and M2, the type of MΦs that is important for implantation is unknown. In this study, we investigated the role of M2 MΦ in implantation using CD206-DTR mice. To deplete M2-MΦ, female CD206-DTR C57/BL6 mice were injected with DT before implantation. These M2-MΦ depleted mice (M2(-)) were naturally mated with Balb/C mice. As the control group, female C57/BL6 wild type (WT) mice injected with DT were mated with male Balb/C mice. The number of implantation sites and plasma progesterone levels at implantation were examined. Implantation-related molecule expression was determined using quantitative-PCR and immunohistochemistry of uterine tissues. The mRNA expression in the endometrial tissues of 38 patients with implantation failure was examined during the implantation window. In WT mice, CD206+M2-like MΦs accumulated in the endometrium at the implantation period, on embryonic (E) 4.5. In M2(-), the implantation number was significantly lower than that in control (*p* < 0.001, 7.8 ± 0.8 vs. 0.2 ± 0.4), although the plasma progesterone levels were not changed. Leukemia inhibitory factor (LIF) and CD206 mRNA expression was significantly reduced (*p* < 0.01), whereas the levels of TNFα were increased on E4.5 (*p* < 0.05). In M2(-), the number of Ki-67+ epithelial cells was higher than that in control at the pre-implantation period. Accelerated epithelial cell proliferation was confirmed by significantly upregulated uterine fibroblast growth factor (FGF)18 mRNA (P < 0.05), and strong FGF18 protein expression in M2(-) endometrial epithelial cells. Further, M2(-) showed upregulated uterine Wnt/β-catenin signals at the mRNA and protein levels. In the non-pregnant group, the proportion of M2-like MΦ to pan MΦ, CD206/CD68, was significantly reduced (*p* < 0.05) and the TNFα mRNA expression was significantly increased (*p* < 0.05) in the endometrial tissues compared to those in the pregnant group. CD206+ M2-like MΦs may be essential for embryo implantation through the regulation of endometrial proliferation via Wnt/β-catenin signaling.

## Introduction

Macrophages (MΦ) are a crucial player in the generation and execution of immune responses through various functions, including phagocytosis, antigen presentation, and secretion of a variety of cytokines and growth factors (1–3). Recently, MΦs have been reported to play an essential role in tissue development and homeostasis through increased angiogenesis and vascular remodeling (1, 4, 5). MΦs also have attracted significant interest in human diseases as they play crucial roles in many diseases associated with chronic inflammation such as atherosclerosis, obesity, diabetes, cancer, skin diseases, and neurodegenerative diseases (6, 7). Implantation is a vital process of the first feto-maternal encounter in the uterus, leading to pregnancy. Good coordination between a blastocyst and receptive uterus is essential for successful implantation (8, 9). Although implantation is an important phenomenon in pregnancy, its precise mechanism is not fully understood due to its complexity involving multi-factors. Animal studies using different kinds of genetically altered mice have been undertaken to elucidate the mechanism of implantation (10–13). Although few studies have examined the relationship between MΦ and implantation, Care et al. first reported that MΦ plays an important role in the implantation process in CD11b-DTR mice (14). They showed that depletion of CD11b+ MΦs resulted in the implantation failure due to decreased progesterone production in the corpus luteum (14). MΦs are classified into two subtypes, M1 and M2 MΦs. M1 MΦs, or classically activated MΦs, are pro-inflammatory and play a central role in host defense against infection, whereas M2 MΦs, or alternatively activated MΦs, are associated with responses to anti-inflammatory reactions and tissue remodeling (15).

The precise role of MΦs in the uterus at the implantation period is unclear in implantation period.

MΦs demonstrate plasticity and polarize to the M1 or M2 type according to their surrounding microenvironment and stimuli (2, 16) and skewness to M1 or M2 MΦs has been reported in various diseases (4). But it is not clear which type of MΦs mostly contributes to the implantation. In the present study, we investigated the role of CD206+ M2-like MΦ in implantation using CD206-diphtheria-toxin (DT)-receptor transgenic mice (17–19), in which M2-like MΦs can be specifically depleted.

## Results

### CD206+M2-Like MΦs Are Located in the Uterus at the Implantation Period

**Figure 1:**
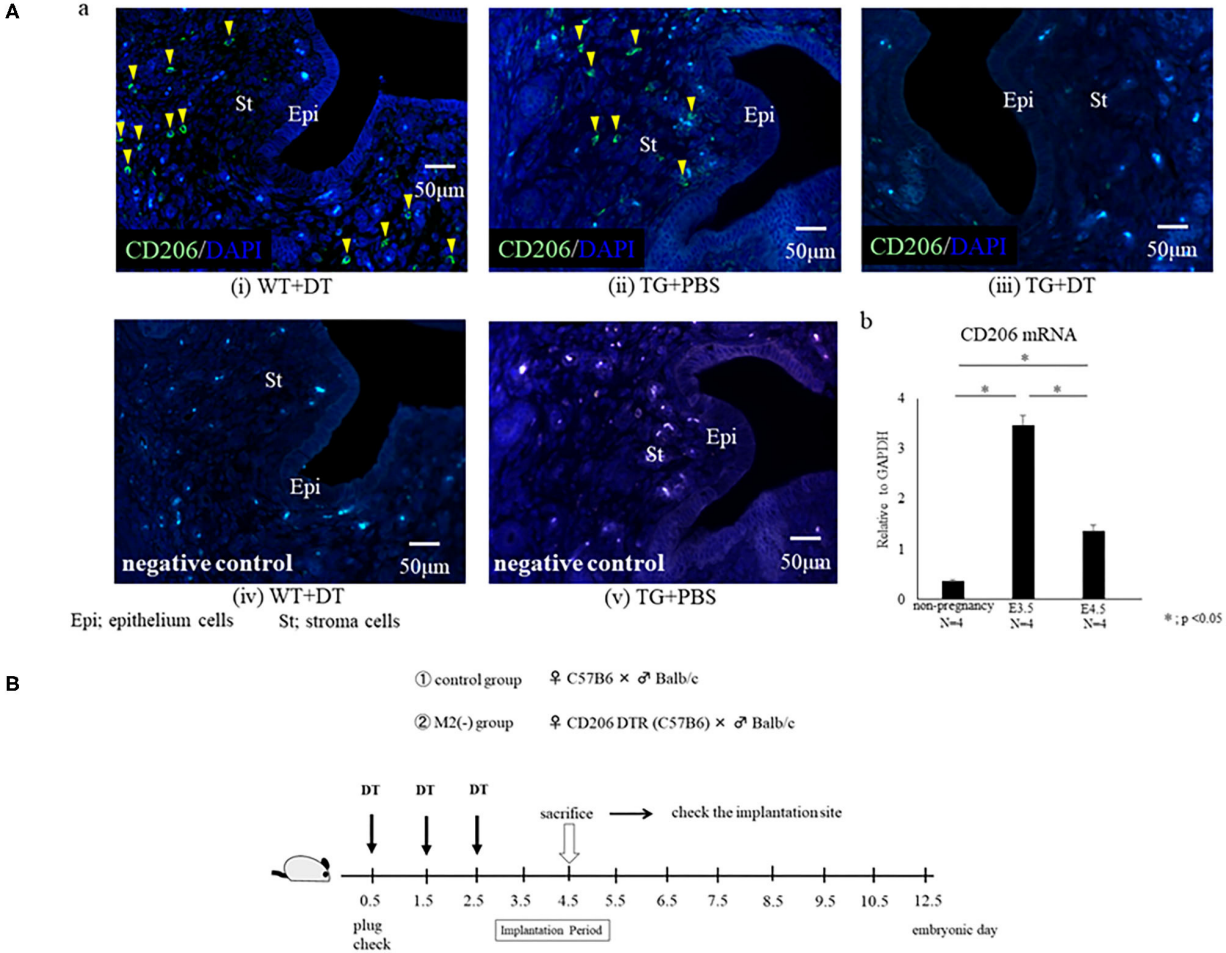
**(A)** Localization of CD206+ M2-like macrophages (MΦ) in the uterus. Immunofluorescence for CD206 was performed for the uterus at embryonic day 4.5 (E4.5) in wild type (WT) and CD206 diphtheria toxin receptor transgenic mice (TG). The data of WT+DT(i), TG+PBS (ii), TG+DT (iii), WT+DT (iv), and TG+PBS(v) were shown. Anti- CD 206 antibody (i, ii, and iii) or control rabbit IgG (iv and v) were used for primary antibody. CD206-positive cells are in green, and nucleus were stained in blue. Yellow arrow heads show CD206+ M2-like MΦ. The uterine mRNA expression of CD206 at non pregnancy, E3.5 and E4.5 are shown in (b). Data were normalized to GAPDH mRNA levels to determine the relative abundance and are shown as the mean ± SEM. *p < 0.05. **(B)** Depletion protocol for CD206+ M2-like macrophages (MΦs) in the implantation model using CD206 DTR mouse. After checking the plug, diphtheria-toxin (DT) was intra-peritoneally injected prior to the implantation period (embryonic day; E0.5, E1.5, E2.5) in CD206-DTR female mice naturally mated with Balb/C male mice, defined as the M2(-) group. DT was also injected to C57/B6BL wild type (WT) female mice mated with Balb/C male mice, and were defined as the control group. The number of implantation sites and plasma progesterone levels at the implantation period (E4.5) were examined.

Most MΦs in non-pregnant mice are known to be present in the uterine stroma, but are reported to exist in the lumen and glands during the implantation period (20). To examine the localization of M2-like MΦs in the uterus at the implantation period on embryonic day 4.5 (E4.5), we performed CD206 immunohistochemistry in wild type (WT) mice. At the implantation period, we found that CD206+ M2-like MΦs were located in the uterine stromal region as well as close to the lumen and glands. Immunofluorescence analysis revealed that CD206+ cells were found in WT with DT and TG with PBS group, while these were completely depleted in TG with DT mice (Figure 1A-a). To examine the change of M2-like MΦs in the uterus at the implantation period, we compared CD206 mRNA expressions between non-pregnancy and implantation periods. The mRNA expressions of uterine CD206 was significantly increased during implantation period, peaking at embryonic (E) 3.5, compared to non-pregnancy (Figure 1A-b).

### Implantation Was Impaired in the M2(-) Group

**Figure 2:**
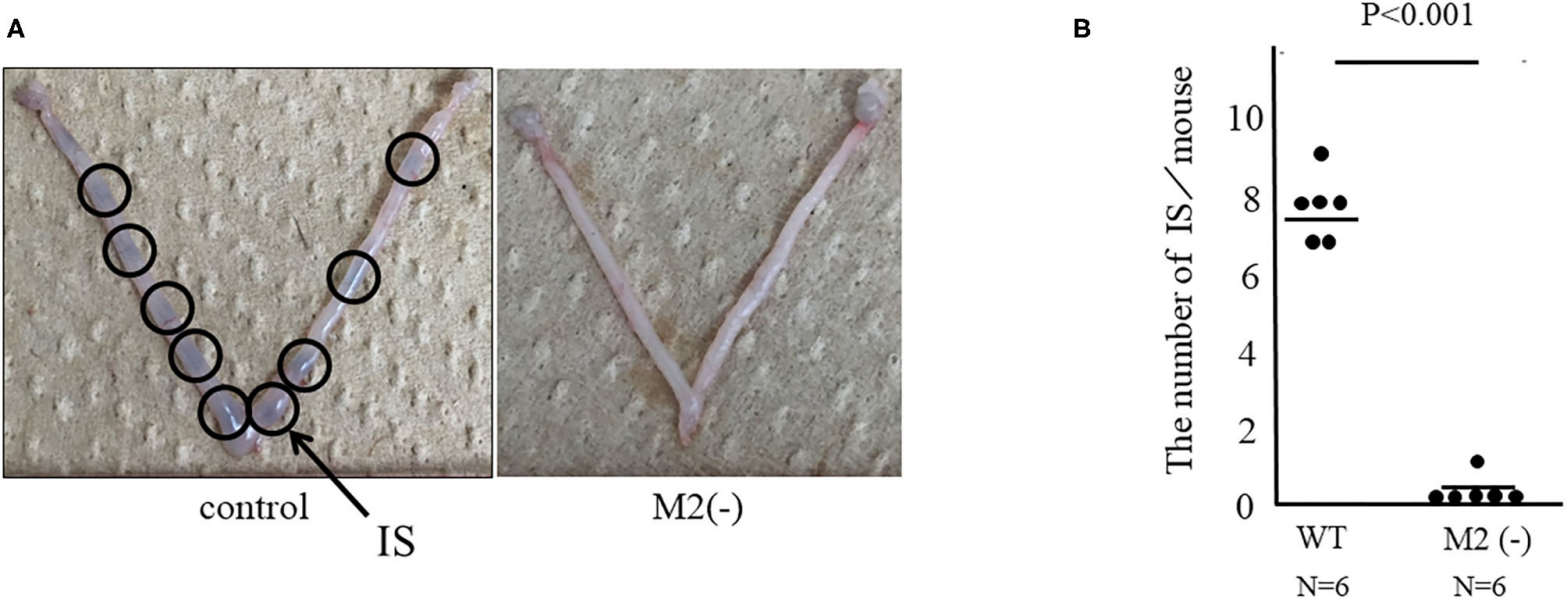
Assessment of implantation site (IS). Representative appearances of the IS in CD206 DTR with DT (M2(-)) and control. ISs were stained with Chicago blue dye for facilitating their detection. ISs are shown (arrows) **(A)** and the number of ISs are shown as dots and the mean **(B)**.

**Figure 3:**
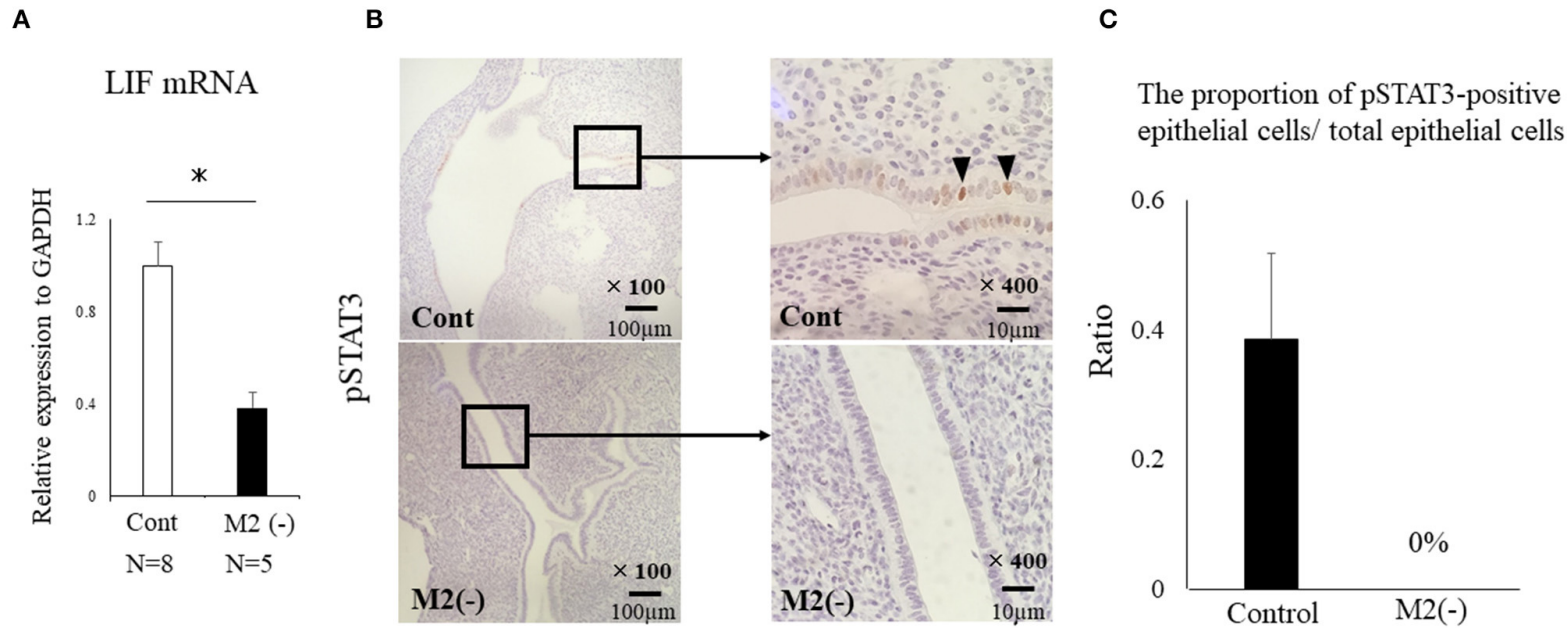
LIF-STAT signal in the uterus at the implantation period. LIF mRNA expression in the uterus was examined. Data were normalized to GAPDH mRNA levels to determine the relative abundance and shown as **(A)**. *p < 0.05. Immunohistochemistry was performed for phosphorylated (p) STAT3 in the control and M2(-) mice **(B)**. We randomly selected six different sites on the section of immunostaining and counted the number of phosphorylation (p) STAT3-positive epithelial cells, which were divided by the total number of epithelial cells between both groups. The proportion of pSTAT3-positive epithelial cells/ total epithelial cells was examined **(C)**.

To investigate the role of CD206+ M2-like MΦ in implantation, we set the protocol of an implantation model using CD206 DTR mice (Figure 1B). We naturally mated C57/B6 female mice with Balb/c male mice as controls, or mated CD206 female DTR mice with Balb/c mice, defined as M2(-). DT was intraperitoneally administered to the control and M2(-) mice before the implantation period. The depletion of CD206+ M2-like MΦs was checked by qPCR. We compared the number of implantation sites between the two groups at E4.5 after in Chicago Blue dye administration. The implantation sites in M2(-) mice were significantly fewer compared to those in the control mice (*P* < 0.001, 7.8 ± 0.8 vs. 0.2 ± 0.4) (Figures 2A,B). As leukemia inhibitory factor (LIF)-Stat signaling is known to be essential for implantation (21), LIF mRNA expression was examined in the uterine tissues in M2(-) mice and was found to be significantly decreased compared to that in control (*p* < 0.05, Figure 3A). Immunostaining for phosphorylated Stat3 was also found in uterine epithelial cells of control mice but not in M2(-) mice (Figure 3B). The proportion of phosphorilated (p) STAT3-positive epithelial/total epithelial cells was significantly reduced in the M2(-) group compared to control (mean ± SD, 38.5 ± 13.2% vs. 0%; *p* < 0.01, Figure 3C).

### The Accelerated Proliferation of Epithelial Cells Was Found in M2(-) Mice

**Figure 4:**
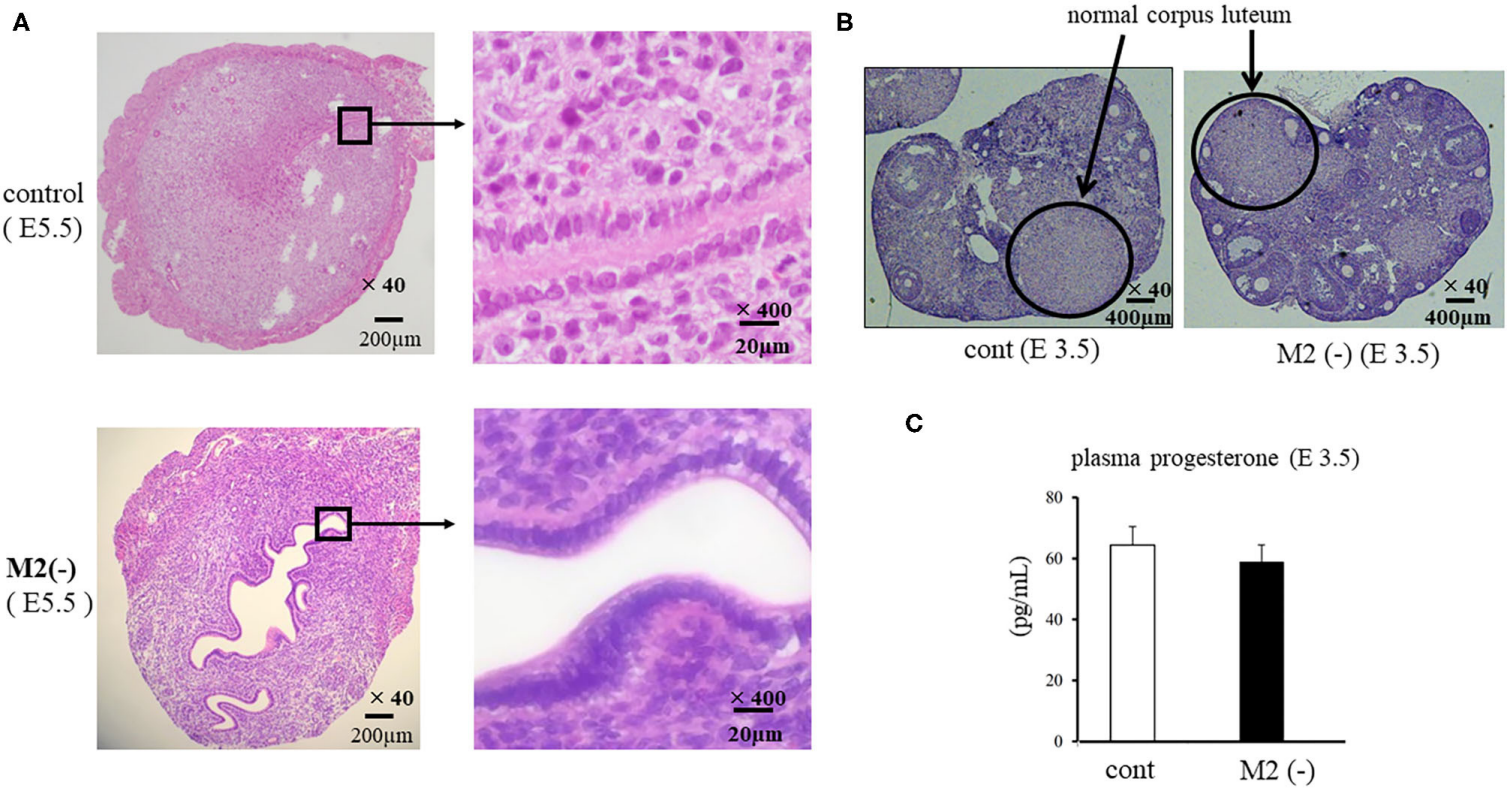
Cross section of uterus and ovary. The cross sections of the implantation site (IS) are stained by hematoxylin and eosin (HE). The representative HE sections are shown at lower (x40) and higher (x400) magnification in the control and M2(-) at E5.5 **(A)**. The corpus luteum [**(B)**, hematoxylin and eosin stain] and the plasma progesterone levels **(C)** in control and M2(-) at E 3.5 are shown.

**Figure 5:**
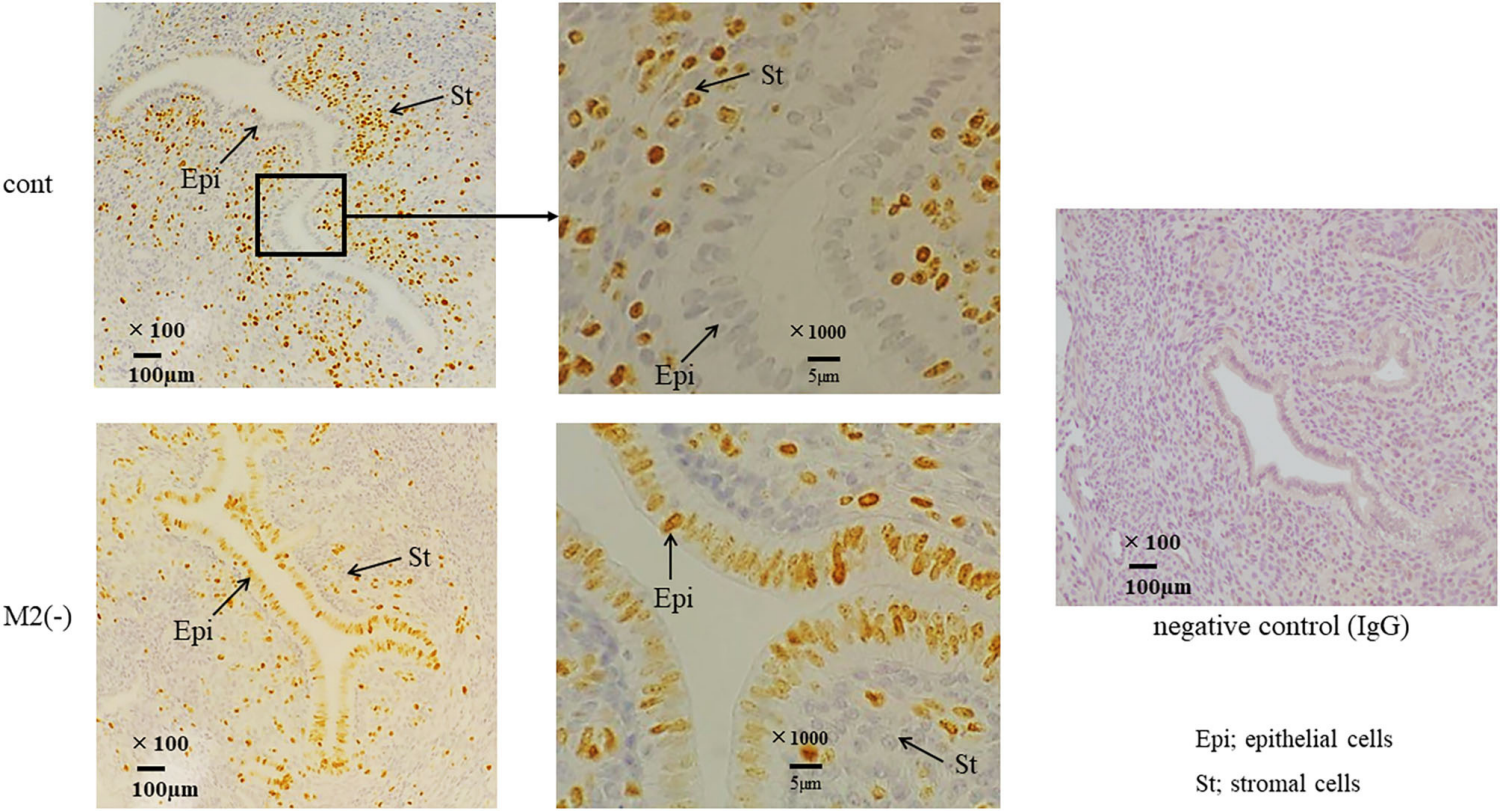
Ki-67 immunostaining in the uterus at the pre-implantation period. Immunostaining for Ki-67, a proliferation marker, was performed in control and M2(-) mice. The arrows indicate the endometrial epithelial and stromal cells at E3.5. The representative sections are shown at lower (x100) and higher (x1000) magnification. Rabbit IgG was used for negative control.

We examined the morphological changes in the just after implantation period (E 5.5). In M2(-) mice, cell proliferation in the stromal region was impaired, and epithelial cells were proliferative compared to control (Figure 4A). We then examined the cell proliferation at pre-implantation period (E3.5). In M2(-) mice, the number of Ki-67-positive epithelial cells was higher compared to that in control at the pre-implantation period (E3.5). However, there were no histological differences in the corpus luteum and the plasma P4 concentration between both groups (Figures 4B,C). These suggest that endometrial epithelial cells had not transformed to become receptive to embryo implantation (Figure 5).

### Uterine Wnt/β-Catenin Signaling Is Upregulated in M2(-) Mice at the Pre-implantation Period (E3.5)

**Figure 6:**
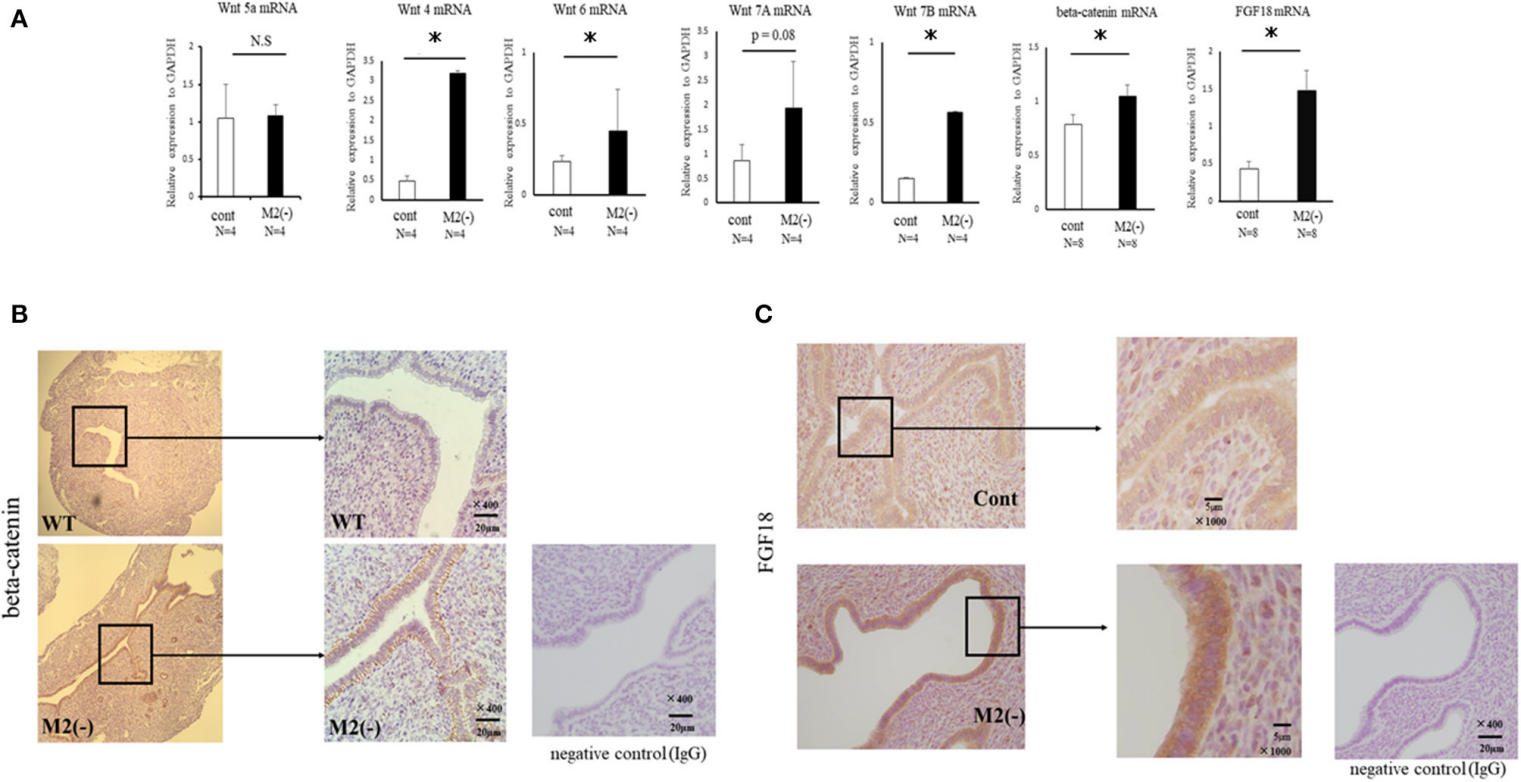
Wnt/β-catenin signaling in the uterus at the pre-implantation period. The mRNA expression of Wnt4, Wnt5a, Wnt6, Wnt7A, Wnt7B, β-catenin, and FGF18 in the uterus at E 3.5 was examined by quantitative-PCR in the control and M2(-) mice **(A)**. Data were normalized to GAPDH mRNA levels to determine the relative abundance. The expression of β-catenin and FGF18 protein in the uterus at the pre-implantation period in control and M2(-) mice at E3.5 was examined by immunohistochemistry. Rabbit IgG was used for negative control **(B,C)**. Data are shown as the mean ± SEM. *p < 0.05.

Uterine Wnt/β-catenin signals regulate the production of fibroblast growth factor (FGF), and proper modification of these signals is essential for implantation (22). In M2(-) mice, at the implantation period, the mRNA expression of Wnt 4A, Wnt 7B, and β-catenin, was significantly increased (*p* < 0.05) compared to the control, and endometrial epithelial cells exhibited strong staining for active β-catenin (Figure 6A). In detail, the basal site of uterine epithelial cells was strongly stained with β-catenin in M2(-) mice (Figure 6B). As expected, the mRNA expression of FGF18, downstream of the Wnt/β-catenin signal, was significantly upregulated (*P* < 0.05) compared to that in control; further, FGF18 protein was also strongly stained in the endometrial epithelial cells of M2(-) mice (Figure 6C).

### Uterine Wnt/β-Catenin Signaling Was Enhanced by Inflammatory M1-Like MΦ

**Figure 7:**
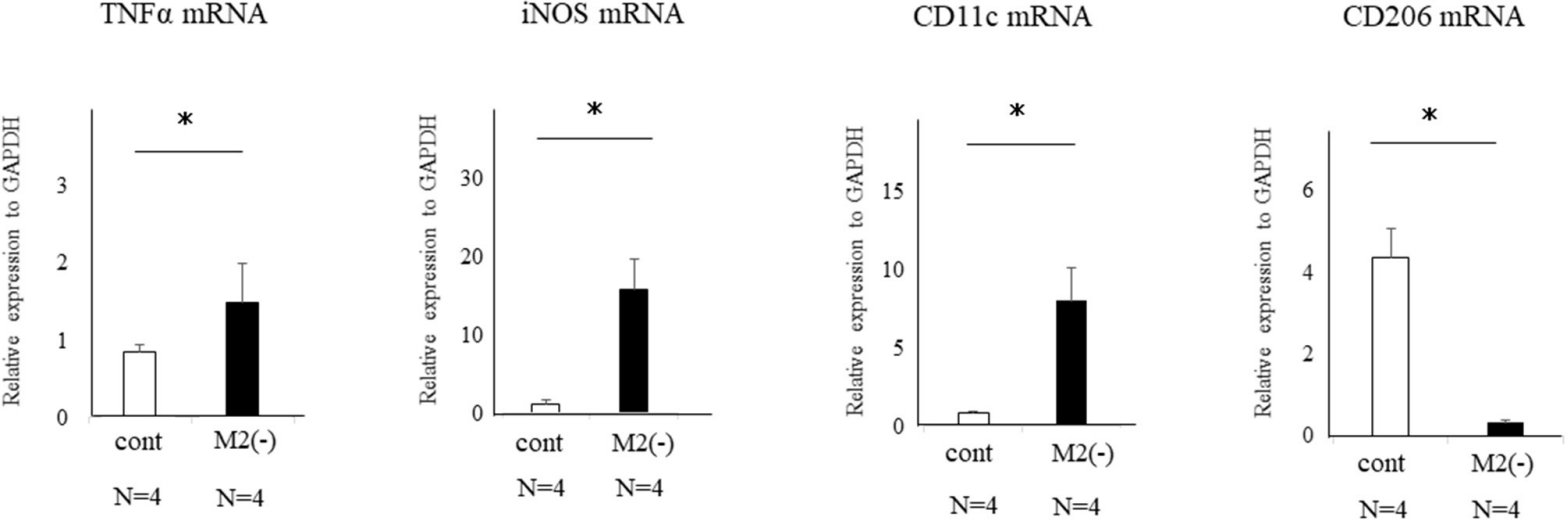
Expression of TNFα, iNOS, CD11c, and CD206-mRNAs in the uterus of control and M2(-) mice at the pre-implantation period (E3.5). The mRNA expression of TNFα, iNOS, CD11c as the M1 MΦ marker, and CD206 in the uterus at the pre-implantation period in control and M2(-) mice was examined. Data were normalized to GAPDH mRNA levels to determine the relative abundance and are shown as the mean ± SEM. *p < 0.05.

Wnt signaling is reported to be increased by TNFα in gastric tumor cells (23). We also found upregulated expression of TNFα, iNOS, and CD11c mRNAs produced by M1-like MΦs (4) in the uterus of M2(-) mice compared to control (*p* < 0.05) (Figure 7).

### The Proportion of CD206+ M2-Like MΦs Among Total MΦs in Uterine Tissues Was Significantly Reduced in Patients With Infertility

**Figure 8:**
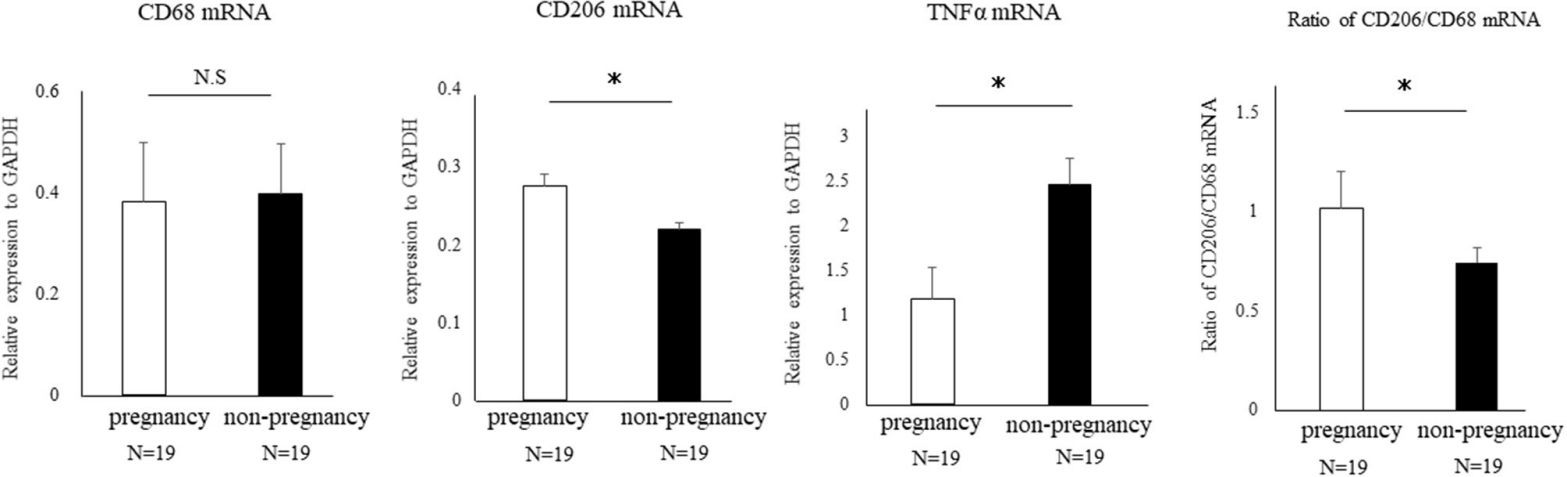
The proportion of uterine CD206+ M2-like MΦ in human cases of pregnancy success and failure. A cohort of 38 patients with implantation failure were subjected to uterine endometrial biopsy at the timing of the implantation window. The mRNA expression of CD68 (pan-MΦ), CD206, and TNFα in uterus were examined between the pregnant and non-pregnant group after endometrial biopsy. Data were normalized to GAPDH mRNA levels to demonstrate the relative abundance. Also, the proportion of uterine CD206+ M2 MΦ to pan-MΦs at the mRNA level between two groups is shown. Data are shown as the mean ± SEM. *p < 0.05.

We performed uterine endometrial biopsy in cohort of 38 infertility patients at the time of the implantation window. Implantation failure was diagnosed as the infertility factor for all these patients. After the endometrial biopsy, 19 patients got pregnant with assisted reproductive technology. The median age of the non-pregnant and the pregnant group was 40 (29–44) years and 37.5 (33–43) years old, respectively, which was comparable between two groups. We then compared the proportion of uterine CD206+ M2-like MΦs to pan MΦs at the mRNA levels of CD206/CD68, between pregnancy and non-pregnant groups. The relative ratio of M2-like MΦ to total MΦ was significantly reduced (*P* < 0.05) in non-pregnant group compared to that in the pregnant group, while upregulation of TNFα mRNA expression was observed in the non-pregnant group (*p* < 0.05) (Figure 8).

## Discussion

This is the first report investigating the role of M2 MΦs in the uterus at the implantation period in the mouse model. Previous reports showed that MΦs are important regulator of implantation and their depletion disrupts luteal vasculature resulting in reduced progesterone production from corpus luteum, which cause implantation failure (14, 24). These data suggest that depletion of pan MΦs during the implantation period causes implantation failure not owing to defects in the uterus but attributed to the ovary. Plaks et al. used the CD11c DTR mouse model to report that uterine dendric cells (DCs) are essential for embryonic implantation (25). However, as CD11c-positive cells include both MΦs and DCs, there was a limitation to determining the effect of each cell on implantation when CD11c cells were depleted. In the present study, we showed for the first time that CD206+ M2-like MΦs are essential to implantation by using CD206 DTR mice.

In the CD206 DTR mouse model, implantation failure occurred exclusively by the depletion of CD206+ M2-like MΦs. As we have reported previously (19), we also found that the histological structure of the corpus luteum in M2(-) was not different from that of the control mice, and the plasma P4 levels were not changed, suggesting that the ovarian function at the implantation period was maintained in the absence of M2-like MΦs. Reduced plasma progesterone level by luteal dysfunction in depletion of pan MΦs mice model might be caused by the depletion of M1 MΦs. Therefore, the implantation failure may be attributed to the abnormal interaction between the embryo and uterus. In our previous study, we examined the effects of oocytes and embryos quality derived from M2MΦ depletion mouse on fertilization and implantation (19). In detail, after inducing superovulation in wild type (WT) and CD206+M2-like MΦ depleted mice, oocytes obtained from the fallopian tubes of these mice were *in vitro* fertilized, followed by transferring to pseudo pregnant WT mice. The fertilization rate, blastocyst formation rate, and pregnancy rate of CD206 DTR-mice derived oocytes were comparable to that of WT-mice derived oocytes, suggesting that oocytes derived from CD206+M2-like MΦ-depleted mice did not affect fertilization and implantation (19). In the present study, the structure of the corpus luteum and the plasma progesterone level was maintained during the implantation period. We detected morphological abnormality only in the uterus, so decreased uterine MΦs were considered the cause of implantation failure. In addition, at the pre-implantation period (E3.5), embryos obtained by flushing the uterine cavity with saline in both WT and CD206 DTR mice exhibited no morphological differences (data not shown). These data suggest that the depletion of M2MΦ *in vivo* did not affect the embryo quality and hormonal milieu. Therefore, the cause of implantation failure in M2(-) mice as observed in the present study, was the uterus and was not due to the abnormalities in the ovary or the embryo. Subsequently, we focused on the role of M2 MΦs in the endometrium during implantation. Elevated P4 concentrations after ovulation dramatically change the state of endometrial cell proliferation and render the uterus receptive to the embryo as a normal uterine morphological change that occurs during pre-implantation (26). In normal conditions, luminal epithelial cells are known to cease proliferation for implantation (26, 27); however, in M2(-) mice, the number of Ki-67-positive endometrial epithelial cells was higher compared to the control at the preimplantation period (E3.5), suggesting that the endometrial epithelial cells did not undergo the required change for receiving the embryo (Figure 5).

Nallasamy et al. have reported that targeted mutation of the homeobox transcription factors, Msx1 and Msx2, which control organogenesis and tissue interactions during embryonic development, in both the uterine epithelium and stroma, results in implantation failure. Based on gene expression profiling of the uterine epithelium and stroma from Msx1/2d/d mice, elevation of Wnt/β-catenin signaling leads to an increase in fibroblast growth factor (FGF) production in the uterine stroma (28). Moreover, upregulated FGFs act in a paracrine manner on the uterine epithelium to promote epithelial proliferation, which prevents endometrial differentiation and creates a non-receptive uterus for the embryo (28). This indicates that an excessive increase in Wnt/β-catenin signaling leads to an unreceptive uterus, which is refractory to implantation due to its inability to control epithelial proliferation, though moderately balanced uterine Wnt/β-catenin signaling is reported to be necessary for implantation (22). In the present study, the accelerated proliferation of epithelial cells in M2(-) mice might be due to a higher expression of FGF-18 in endometrial epithelial cells.

**Figure 9:**
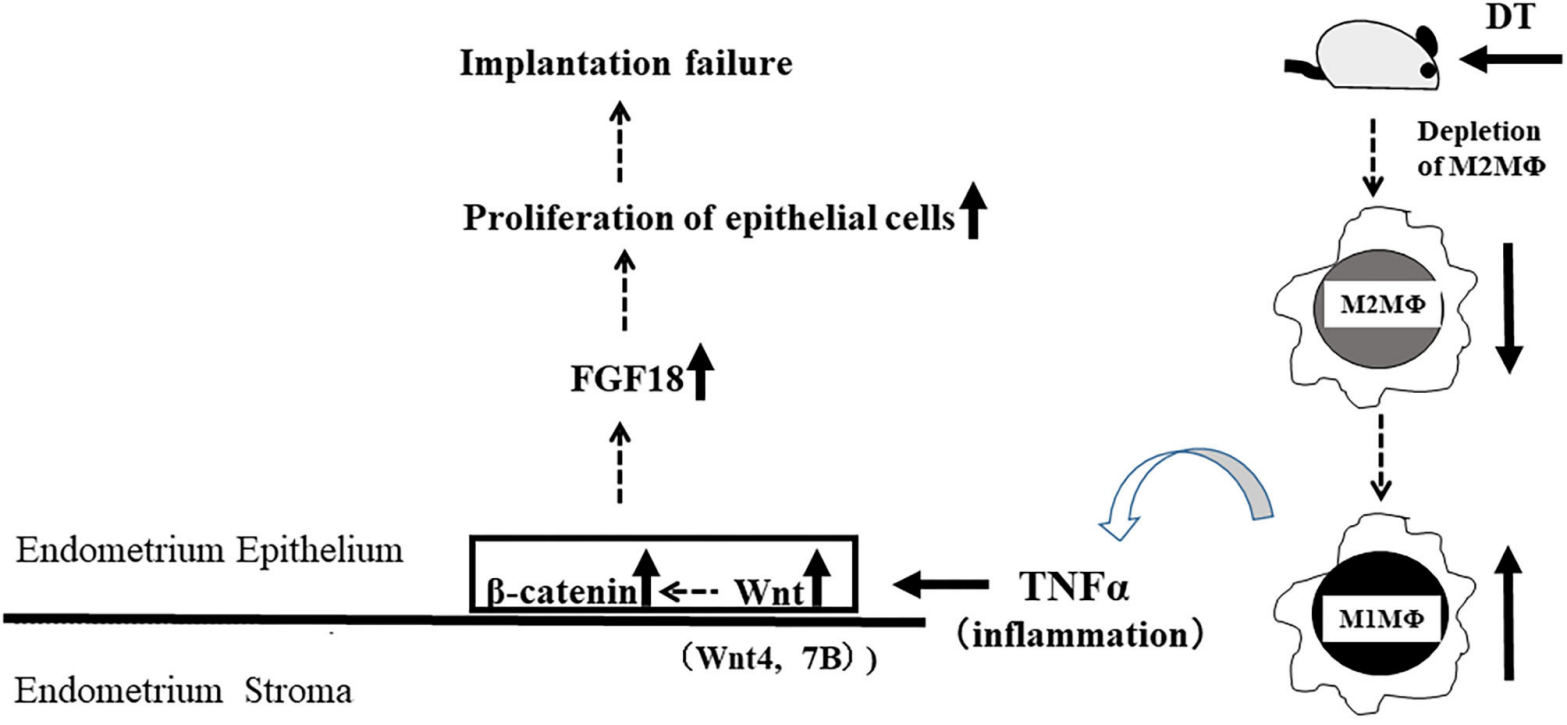
Schematic of the study.

Aberrant expression of TNFα has been reported as one of the causes of enhanced Wnt/β-catenin signaling (23). In the present M2(-) mouse model, we found that the expression of TNFα and M1-like MΦ markers such as inducible nitric oxide synthase (iNOs) and CD11c (10), were significantly increased at the mRNA level (*P* < 0.05). This upregulation of M1-like MΦ related molecules might be due to a relative increase in M1-like MΦs owing to the depletion of CD206+ M2-like MΦs. Kambara et al. also reported that the expression of pro-inflammatory cytokines such as TNFα, IL-1β, IL-6, and MCP-1 were significantly upregulated in the lung tissues of CD206 DTR mice in response to DT treatment (17). Collectively, we hypothesized that upregulation of TNFα secreted by M1-like MΦs after depletion of CD206+ M2-like MΦ might accelerate the uterine Wnt/β-catenin signal in endometrial epithelial cells. Further, in epithelial cells, FGF18 expression was increased aberrantly, resulting in the proliferation of epithelial cells, which caused implantation failure (Figure 9). These results indicate that the balance of M1 and M2 MΦs may be critical for embryonic implantation. Additionally, LIF, essential for implantation, is also known to be produced by MΦ (29) and MΦ derived LIF is identified as a potential factor mediating MΦ-epithelial signaling (30). Therefore, the decrease in LIF expression (Figure 3) might be involved in the implantation failure. In our analysis of human samples, the proportion of uterine CD206+ M2-like MΦ, based on the CD206 mRNA expression compared to the total MΦ marker CD68, was significantly reduced (*P* < 0.05) in non-pregnant patients at the implantation period compared to that in the pregnant patients. And, in non-pregnant patients, the TNFα mRNA expression was significantly increased compared to that in the pregnant patients. Thus, dysregulation of M1/M2 MΦs may be one of the causes of implantation failure in humans.

In conclusion, we showed that the depletion of M2 MΦ led to implantation failure. Further studies are needed to clarify whether the mechanism of implantation failure is due to change in balance of M1/M2 MΦs, or decrease in number of M2 MΦs.

## Materials and Methods

### Reagents and Materials

Roswell Park Memorial Institute (RPMI)-1640 medium and Diphtheria Toxin (DT) were purchased from Sigma-Aldrich (St. Louis, MO, USA). Fetal bovine serum (FBS) was purchased from Life Technologies (Minato-ku Tokyo, Japan). Antibiotics (a mixture of penicillin, streptomycin, and amphotericin B) were purchased from Wako Pure Chemical Industries (Chuo-ku, Osaka, Japan).

### Immunohistochemistry

Paraffin-embedded tissues were cut into 5-μm-thick sections and mounted on slides. The mouse uterine and ovarian sections were deparaffinized in xylene, rehydrated through a graded series of ethanol, and washed in water. Antigen retrieval was performed in 10 mM sodium citrate buffer (pH 6.0) in a microwave for 10 min and then cooling to room temperature. Rabbit IgG was used as a negative control. Slide staining with the first and second antibodies was performed according to the manufacturer's instructions. Immunostaining was performed using antibodies specific to Ki-67 (Abcam, Tokyo, Japan, Cat# 15580, 1:100 dilution), β-catenin (Abcam, Tokyo, Japan, Cat# 138378, 1:100 dilution), phosphorilated-STAT3 (Cell Signaling Technology, Massachusetts, USA, Cat #9145, 1:100 dilution), and FGF18 (Abcam, Cat# ab169615, 1:100 dilution). An immunofluorescence analysis of implantation site (uterus) was performed using rabbit anti-mannose receptor (CD206) (Abcam, Cat# 64693, 1:100 dilution). And the primary antibody was incubated overnight at 4°C. As a second antibody, the rat anti-rabbit antibody was used. 4′,6-diamidino-2-phenylindole (DAPI; 1:500) was used to detect nuclei. Rabbit IgG were used instead of the primary antibody for negative control.

### Mice and Diphtheria Toxin Administration

Female, CD206 DTR mice (17, 18), aged 12 to 20-weeks old were used. The mice were housed in a specific pathogen free (SPF) animal facility with a controlled environment of 22–24°C and 60–70% relative humidity, on a 12 h light/12 h dark cycle with food and water provided *ad libitum*. DT was diluted with sterile phosphate buffered saline (PBS) to the desired concentration and was intra-peritoneally injected to mice to deplete the CD206 positive cells. According to BioGPS, a complete resource for learning about gene and protein function (http://biogps.org/), CD206 seems to be expressed in mouse uterus more than other M2MΦ markers. In our preliminary experiment, in each organ, CD206 mRNA seemed to be more expressed than other M2MΦ markers (data not shown). Decidual MΦs are reported to be show higher expression of CD206 (31, 32). Wang et al. (33) reported that the CD206 expression in MΦs could be a marker for spontaneous abortion. From these results, CD206 is considered to be a valid marker for uterine M2 MΦ. The experiments and procedures were performed at 48 h after the final DT administration, as previously reported by Nawaz et al. (18). The final DT injection was administered at E 2.5 before implantation. The depletion of CD206 positive cells was confirmed at mRNA levels by qPCR every experiment.

### Assessment of the Implantation Site (IS)

We prepared control group by mating C57/B6 female WT mice with Balb/c male mice, and M2(-) group by mating CD206-DTR female mice with Balb/c mice (Figure 1B). To deplete M2-like MΦs at the implantation period, DT was administered to each mouse at a dose of 30 ng/gram body weight before implantation. We then checked the implantation sites between two groups. To identify implantation sites on embryonic day 4.5 (E4.5), mice were anesthetized using Avertin (2% tribromoethanol, 15 μl/g i.p.; Sigma-Aldrich), administered the Chicago blue dye solution (0.4% in PBS i.v.; Sigma-Aldrich) and then analyzed after 10 min. Uteri were dissected and assessed for clearly delineated blue bands as evidence of early implantation sites. In other mice, uterine paraffin sections from control and M2(-) mice were collected on E3.5 and E4.5 and stained with H&E to assess the implantation sites.

### Patients With Implantation Failure

Uterine endometrial biopsy as performed at the time of the implantation window in 38 patients with implantation failure who visited the outpatient department of obstetrics and gynecology at the University of Tokyo. After the endometrial biopsy, we compared the proportion of uterine M2-like MΦ to the pan-MΦ based on the mRNA levels of CD206 and CD68 between the pregnancy and non-pregnant group.

### Reverse Transcription (RT) and Quantitative Real-Time Polymerase Chain Reaction (PCR) Analysis

Total RNA was extracted from the mouse endometrial region from the peritoneal cavity, using the ISOGEN-II (NIPPON GENE, Tokyo, Japan). RT was performed using Rever Tra Ace qPCR RT Master Mix with gDNA Remover (TOYOBO, Tokyo, Japan). About 1.0 μg of total RNA was reverse-transcribed in a 20-μL volume. For the quantification of various mRNA levels, real-time PCR was performed using the Mx3000P Real-Time PCR System (Agilent Technologies, CA, USA) according to the manufacturer's instructions. The PCR primers used with the SYBR Green protocol were selected from different exons of the corresponding genes to discriminate the PCR products that might arise from possible chromosomal DNA contaminants. The SYBR Green thermal cycling conditions were as follows: 1 cycle of 95°C for 30 s, and cycles of 95°C for 10 s, 60°C for 10 s and 72°C for 10 s. The primer sequences used were as follows: 3-phosphate dehydrogenase (GAPDH, NM_002046: 628–648 and 1079–1060), mouse CD206 (NM_000710.3: 326–347 and 495–473), mouse TNFα (NM_000623.3: 432–453 and 605–584), mouse IL-10 (NM_010548.2: 390-412 and 464-443), mouse CD11c (NM_001363985.1: 82-103 and 194-175), mouse iNOS (NM_001313922.1: 2363-2382 and 2489-2470), β-catenin (NM_000623.3: 432–453 and 605–584), Fibroblast Growth Factor 18 (NM_000623.3: 432–453 and 605–584), Wnt 4 (NM_009523.2: 318-337 and 426-409), Wnt 5A (NM_009524.4: 565-583 and 668-650), Wnt 6 (XM_006495889.2: 670-688 and 796-779), Wnt 7A (NM_001363757.1: 501-518 and 578-558), and Wnt 7B (NM_009528.3: 421-440 and 492-472). The relative mRNA levels were calculated using the standard curve method and were normalized to the mRNA levels of GAPDH (forward, 5′-AATGTGTCCGTCGTGGATCTGA-3′ and reverse, 5′-GATGCCTGCTTCACCACCTTCT-3′) (Supplementary Data 1).

### Measurement of Estradiol (E2) and Progesterone (P4) Levels

Mouse blood samples were collected during the analysis. Plasma levels of E2 and P4 were measured in duplicate using the specific EIA kits (Cayman, USA).

### Statistical Analysis

Data were evaluated by Mann Whitney test using Jump version 10. *P* < 0.05 was accepted as statistically significant.

## Data Availability Statement

The datasets generated for this study can be found in online repositories. The names of the repository/repositories and accession number(s) can be found in the article/Supplementary Material.

## Ethics Statement

The studies involving human participants were reviewed and approved by the committee of the University of Tokyo (10991). The patients/participants provided their written informed consent to participate in this study. The animal study was reviewed and approved by the committee of University of Toyama (A 2015med-55).

## Author Contributions

SS, OY, KT, YH, and YOn: conception and design. YOn, ANak and AU: acquiring and processing samples. YOn, ANaw, YF, AU, OY, and ES: execution of the experiment. YOn and OY: analysis of data. SS, OY, ANaw, TH, ANak, and YOs: interpretation of data. YOn and OY: drafting the manuscript. SS, OY, YOn, and SW: revision of the manuscript for important intellectual content. All authors contributed to the article and approved the submitted version.

## Conflict of Interest

The authors declare that the research was conducted in the absence of any commercial or financial relationships that could be construed as a potential conflict of interest.

## Supplementary Material

Supplementary Data 1 Primer sequences of mouse and human.
