# Supplementary figures and images for: CD206+ M2-Like Macrophages Are Essential for Successful Implantation
Source: Front Immunol. 2020 Oct 23;11:557184. doi: 10.3389/fimmu.2020.557184 (PMC7644510; doi:10.3389/fimmu.2020.557184)

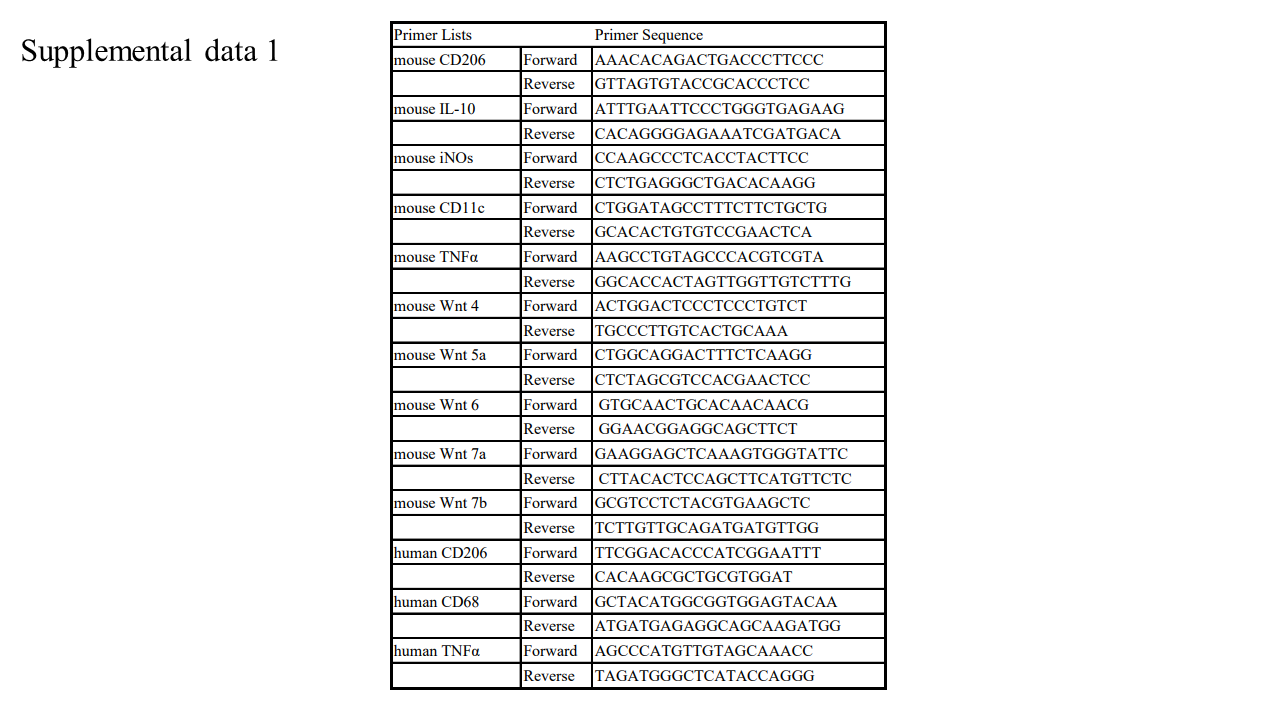

Supplement: Supplementary Data 1 — Primer sequences of mouse and human. [file Image_1.TIF]
